# Editorial: Proceedings of ISPMF 2018 - Plant Molecular Farming

**DOI:** 10.3389/fpls.2020.00492

**Published:** 2020-04-28

**Authors:** Anneli Ritala, Heiko Rischer, Suvi Tuulikki Häkkinen, Jussi Joonas Joensuu, Kirsi-Marja Oksman-Caldentey

**Affiliations:** VTT Technical Research Centre of Finland Ltd., Espoo, Finland

**Keywords:** plant molecular farming, recombinant protein, biopharmaceutical, *Nicotiana benthamiana*, glycosylation

Plant Molecular Farming Research Topic was launched in the 3rd Conference of the International Society of Plant Molecular Farming (ISPMF) in Helsinki, Finland, on June, 2018. Altogether, this Research Topic attracted 31 manuscripts of which 23 were accepted and published. The articles cover recent outcomes and success stories in Plant Molecular Farming and some of the highlights are summarized below.

Knödler et al. reported an important finding related to production of recombinant proteins in tobacco plants in greenhouse conditions. Their data indicates that high temperatures (>28°C) and intense illuminance (>45 klx h−1) may cause substantial loss in the target protein yield due to stability problems. They showed that up to 90% of the product can get lost because of the extreme conditions. This high instability caused by the environmental factors needs to be taken into consideration when designing the contained production facilities for recombinant proteins. It might even be that a fully-controlled indoor farm turns out to be the most cost effective choice.

The paper of Goulet et al. describes the largely neglected phenomena how the basic culture conditions can substantially influence on growth and overall performance of plants producing recombinant pharmaceuticals. They used transient protein expression system using *N. benthamiana* and a promising vaccine antigen, influenza hemagglutinin H1, as a model. It was demonstrated that H1 antigen is not evenly distributed in the plant. The production yields were highly influenced by the age of plant leaves, young leaves being better producers than older ones. The auxillary stem leaves contributed more than 50% of total yield of antigen even though representing less than 30% of the total biomass.

Protein properties such as folding, structure, and function are substantially affected by N-glycosylation. Therefore, especially glycoprotein-based therapeutics rely on optimal N-glycosylation patterns. Xiong et al. produced three variants of the anthrax decoy protein rCMG2-Fc, an antitoxin resulting from the fusion of receptor Capillary Morphogenesis Gene 2 protein with the salvage neonatal Fc-receptor, in *Nicotiana benthamiana* plants. Notably, the authors show that all variants were able to bind the protective antigen of the anthrax toxin. Expression, integrity and thermostability were, however, differentially affected by glycosylation.

Soluble envelope (Env) glycoproteins constitute promising antigens for human immunodeficiency virus type 1 (HIV-1) vaccine development. Margolin et al. report the development of an *Agrobacterium*-mediated transient expression system for the production of cognate soluble HIV-1 subtype C gp140 antigens in *N. benthamiana* as an alternative for conventional production in mammalian cells, which is costly and limited in scalability. They present the successful production of trimeric soluble HIV-1 Env protein although with low yields of ~5–6 mg/g fresh weight of purified protein and demonstrate promising immunogenicity in rabbits.

Nanotechnology is a rapidly advancing field applying nanostructured materials from various organic and inorganic sources. Applications in the field of vaccination, electronics, and bioimaging have been developed and especially for the latter, of PVX particles show unforeseen potential. They are able to carry large payloads and due to their filamentous structure tumor homing and retention properties are better than those of spherical structures. In addition, being protein-based nanoparticles, they are more suitable for biomedical applications compared to their synthetic competitors. A review by Röder et al. gathers technologies applying PVX nanoparticles and describes future opportunities and challenges for the utilization of PVX nanoparticles in various scientific fields.

Food-related bacterial outbreaks are occurring with increasing frequency and severity, enhanced by globalization of food production and active transportation of food ingredients and products. In addition, increasing interest by consumers for “organic” foods result in avoidance or reduction of chemicals and antibiotics by farmers and processing industry, leading to practices which may introduce additional risks of bacterial contamination. Hahn-Löbmann et al. describe the use of plant-made recombinant proteins namely colicins and salmocins in food processing applications. Nomad Bioscience, using the GRAS (Generally Recognized As Safe) regulatory process in the United States, has obtained favorable regulatory review and marketing allowance from the FDA for its *Escherichia*-derived antibacterial proteins, colicins, for the food use. Salmocins—colicin-type proteins derived from *Salmonella*—are currently under GRAS status approval. The research and development, as well as regulatory and economic aspects of both compounds as food antimicrobials are discussed.

Human papilloma virus (HPV) tumor disease causes major health risks especially in developing countries. Massa et al. used tomato hairy roots to produce therapeutic vaccines against HPV, expressing a harmless form of the HPV type 16 E7 protein fused to a non-cytotoxic form of the saporin protein. Hairy root clones were obtained by infecting leaves of *Solanum lycopersicum* yielding approximately 35.5 μg/g of fresh biomass of expressed protein. Immunological response associated to anticancer activity was shown and in particular, synergistic effect of using DNA as prime, and hairy root extract as boost demonstrated the highest efficacy. In this work, the possibilities for using hairy root technology as plant-based biofactories was described, showing the great potential for biomedical applications.

Schillberg et al. presented a critical review on the plant-based expression systems including the highlights and bottlenecks of the platform. The most obvious obstacles are remaining low yields and challenges for the downstream processing i.e., purification of the product from the plant matrix. The most prominent opportunities of the plant expression systems being better or altered protein functionality and the speed of the transient expression systems. Increasing consumer demand for the animal-free and more “natural” products can be also seen as an asset of the system. The kind of healthy realism presented in the review is most welcome when we prepare ourselves for the future. More commercially viable examples are needed to drive the plant-based production further.

The importance of the Plant Molecular Farming Research Topic is clearly seen in the statistics. Only in a year's time, after the first accepted paper, the Research Topic has gained more than 50 000 views and 7,000 downloads. The 4th ISPMF conference was planned to take place in Rome, Italy on June 8–10, 2020. Unfortunately, the COVID-19 outbreak forced us to cancel the meeting and postpone it to the future. We are convinced that we will get together again and at that time we are having a great 4th ISPMF conference with excellent presentations showcasing outstanding data of the latest breakthroughs of Plant Molecular Farming. Stay tuned at http://www.ispmf.org/.

## Author Contributions

All authors listed have made a substantial, direct and intellectual contribution to the work, and approved it for publication.

## Conflict of Interest

The authors declare that the research was conducted in the absence of any commercial or financial relationships that could be construed as a potential conflict of interest.

